# Magnetic Resonance Enterography: The Test of Choice in Diagnosing Intestinal “Zebras”

**DOI:** 10.1155/2015/206469

**Published:** 2015-01-26

**Authors:** Anjali S. Kumar, Jasna Coralic, Reid Vegeler, Kirthi Kolli, John Liang, Allison Estep, Allen P. Chudzinski, James D. McFadden

**Affiliations:** ^1^Section of Colon and Rectal Surgery, Department of Surgery, MedStar Washington Hospital Center, Washington, DC 20010, USA; ^2^Department of Surgery, MedStar Washington Hospital Center, Washington, DC 20010, USA; ^3^Department of Pathology, MedStar Washington Hospital Center, Washington, DC 20010, USA; ^4^Georgetown University School of Medicine, Washington, DC 20057, USA; ^5^Department of Surgery, MedStar Georgetown University Hospital, Washington, DC 20057, USA; ^6^Department of Radiology, MedStar Washington Hospital Center, Washington, DC 20010, USA

## Abstract

Small bowel tumors and other rare intestinal disorders are often exceedingly difficult to identify. Even cutting-edge technologies, such as push enteroscopy and capsule endoscopy, can fail to determine the cause of a patient's symptoms. At our institution magnetic resonance enterography (MRE) has become an increasingly reliable tool in the difficult-to-diagnose or difficult-to-monitor patient. In this retrospective case series, we discuss four patients with four rare intestinal disorders that were successfully diagnosed using MRE after failing to be diagnosed using more routine technologies, such as CT scans and flexible sigmoidoscopies. With the discussion of these four cases we demonstrate that MRE is a useful diagnostic modality in patients whose surveillance is difficult or to diagnose rare colorectal disease phenomena, colloquially referred to as “zebras.”

## 1. Introduction

Magnetic resonance enterography (MRE) is now the preferred imaging modality in inflammatory bowel diseases. MRE has been extensively described in the diagnosis and surveillance of Crohn's disease complications [1, 2]. MRE in the setting of Crohn's disease is especially helpful because repeated computerized tomography (CT) scans subject patients to unacceptably high cumulative radiation [3].

Small bowel tumors and other rare intestinal disorders are often exceedingly difficult to identify. While we can access the stomach, esophagus, duodenum, colon, and rectum by endoscopy, the small bowel has remained elusive to direct visualization. MRE is preferred over capsule endoscopy in identification of polyps in patients with Peutz-Jeghers Syndrome (PJS) [4]. Given its greater capabilities in imaging soft tissues, MRE is especially useful in detection of small bowel lymphomas and can even help determine the histologic diagnosis [5]. Case reports have also demonstrated the importance of MRE in diagnosing other rare disorders, such as* Taenia saginata* and small bowel gastrointestinal stromal (GIST) tumors [6, 7]. Because even cutting-edge technologies such as push enteroscopy and capsule endoscopy can fail to determine the cause of a patient's symptoms, MRE has become an increasingly reliable tool in the difficult-to-diagnose or difficult-to-monitor patient. At our institution, we have noticed that the use of MRE under a highly specific protocol is replacing the use of capsule endoscopy or is called upon when traditional endoscopy and imaging fail.

In this study, we aimed to describe a series of patients with rare intestinal diseases whose diagnoses were obtained with the aid of MRE.  Table 1 shows the summary of the four patients.

## 2. Materials and Methods

Our institutional review board granted the approval for a clinical database that captured data related to MRE tests done at our hospital. From this prospectively collected database of 83 MREs in 66 patients, we retrospectively identified four patients with rare intestinal disorders. We then conducted a detailed chart review of the cases selected.

At our institution, radiologists and technicians have developed a very specific protocol under which MRE is obtained. In short, the protocol involves oral intake of water with a psyllium fiber supplement (Metamucil, Procter & Gamble, Cincinnati, OH) as an intraluminal agent to distend the small bowel prior to examination. The psyllium supplement consists of two teaspoons of Metamucil/450 mL of water repeated four times over a three-hour period. Intravenous glucagon (1.0 mg) is administered to eliminate bowel peristalsis while 15 mL of intravenous gadolinium is used as a contrast agent. Pulse sequences were obtained on MRI machines with 1.5 tesla magnets. The MRE for case one was done with a Philips Intera (Philips, Andover). A Toshiba Titan was the MR machine used in case two while a Siemens Avanto was used for the study in case three. We have found no difference in the quality of exam between MRI machines.

## 3. Results

### 3.1. Case One

#### 3.1.1. History

A 53-year-old male with seven months of crampy abdominal pain, melena, and weight loss of 22 kg was experiencing worsening pain with nausea and vomiting. During the duration of these symptoms he had been evaluated at two different hospitals. Escalating symptoms brought him to seek care from our institution.

#### 3.1.2. Interventions

**Figure 1:**
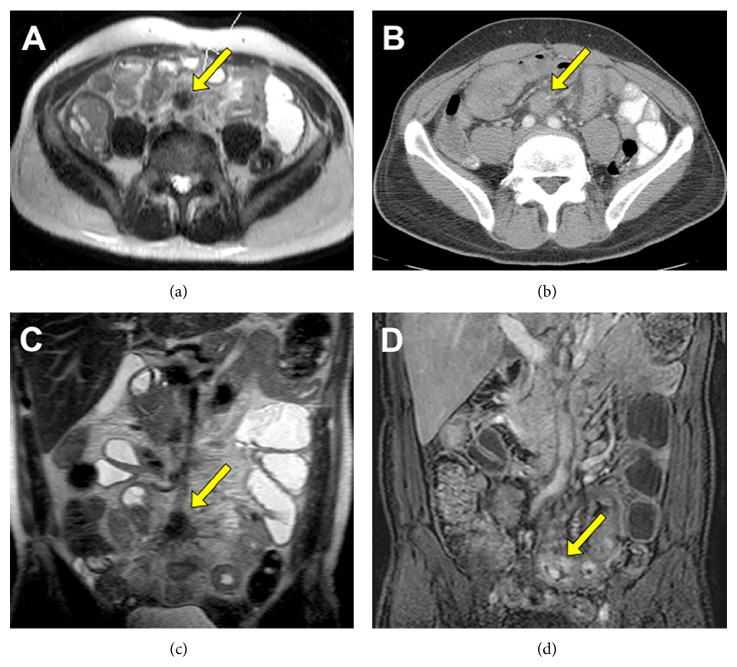
Axial MRE image showing mass in the small bowel mesentery and an enlarged lymph node (a) compared to an axial CT scan that only shows bowel wall thickening (b). Coronal MRE images with carcinoid tumor and an enlarged lymph node in the mesentery (c) and mass in the bowel wall (d).

The patient had multiple repeat endoscopies at these separate institutions as well as a small bowel enteroscopy and CT and MRI scans in the weeks and months prior to the MRE. All of these tests were unable to uncover the etiology for the patient's symptoms. MRE was then pursued, revealing a mass in the small bowel mesentery that was consistent with a carcinoid tumor (Figure 1). The patient was able to proceed to the operating room four days later to have a laparotomy with a small bowel resection and an appendectomy. The finding at the time of surgery was a carcinoid tumor in the distal ileum with invasion into the mesentery.

#### 3.1.3. Diagnosis

**Figure 2:**
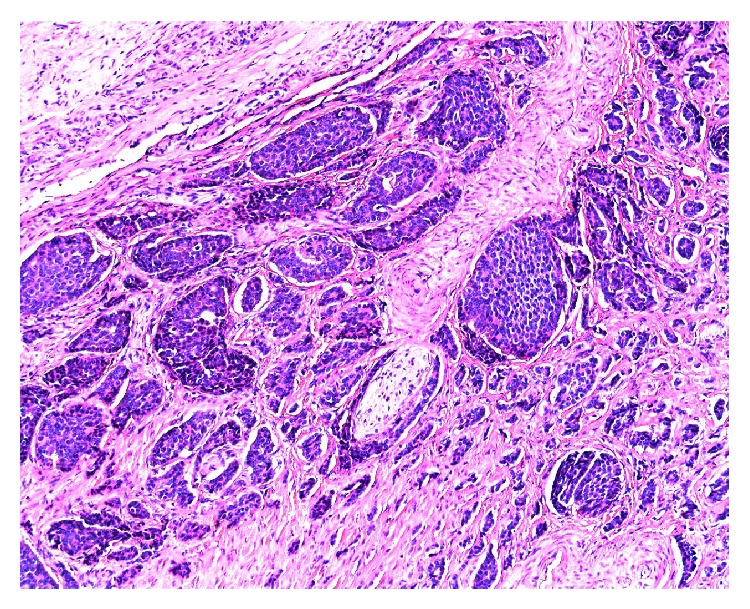
Small bowel carcinoid tumor invading into the subserosal tissue at 20x magnification.

The patient was diagnosed with a small bowel carcinoid tumor with nodal metastasis. Pathology confirmed that the tumor invaded through the muscularis propria with the suspicion of penetration into the peritoneal space (Figure 2). The final pathologic diagnosis was stage IIIB, T4N1M0, small bowel carcinoid tumor. He was sent home on postoperative day eight. Patient did well postoperatively and is alive at the time of this paper's preparation.

### 3.2. Case Two

#### 3.2.1. History

A 34-year-old HIV positive male was seen urgently in our colorectal surgery clinic after a colonoscopy showed an inflamed mucosa and polypoid rectal mass that was partially obstructing. The patient's symptoms had begun as rectal pain six months prior to his visit to the surgery clinic that was soon associated with mucous drainage from his rectum every several days with normal bowel movements in between those. In the months preceding the surgical visit, the patient had been seen by two gastroenterologists and underwent two colonoscopies, the first of which showed inflammation and ulceration of the sigmoid mucosa and the second was the one previously mentioned. He had been started on steroids and narcotics in addition to his other meds, including a clinical trial medication for his HIV diagnosis. His viral load was undetectable and he had a CD4 count in the 200 s.

#### 3.2.2. Interventions

Prior to the patient's clinic visit, he had undergone a colonoscopy, which showed an area of ulceration in the sigmoid colon. Pathologic evaluation of that lesion revealed a benign, nonspecific inflammatory process. In the colorectal surgery clinic the patient underwent anoscopy and rigid sigmoidoscopy, which revealed a nodular and inflamed mucosa but patent rectal lumen. The patient was then scheduled for an MRE.

#### 3.2.3. Diagnosis

**Figure 3:**
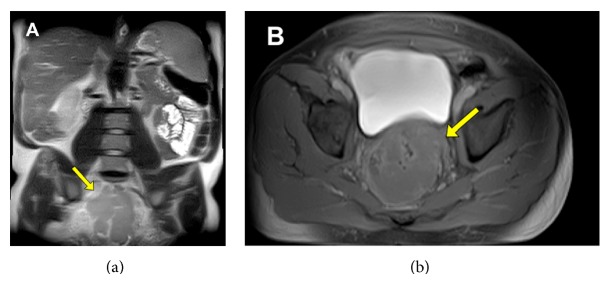
Coronal (a) and axial (b) MRE images demonstrating massive rectal lymphoma and severe narrowing of the rectal lumen (yellow arrows).

**Figure 4:**
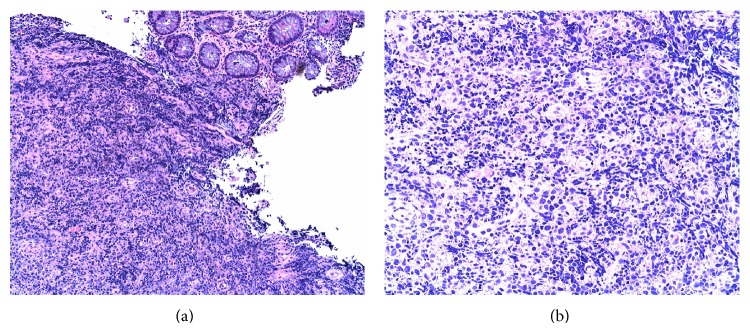
Rectal lymphoma. There is a dense lymphocytic population within a fibrous and vascular stroma. Unremarkable colonic mucosa can be seen in the upper right hand side (10x) (a). The population consists of atypical medium to large lymphocytes with prominent nucleoli and immature chromatin. There are scattered plasma cells and mature lymphocytes present (20x) (b).

The MRE showed significant submucosal inflammation, suggestive of rectal lymphoma with severe, but nonobstructing, lumenal narrowing (Figure 3). The tumor measured 4.3 cm in its maximal diameter and showed evidence of extension through the mesorectal fat to the mesorectal fascia posteriorly. The patient eventually went on to receive chemotherapy after his oncologist obtained confirmatory biopsies (Figure 4) done by the colorectal surgery service via a transanal approach. To this date, the patient has received a full course of chemotherapy and is asymptomatic. He is to have a follow-up imaging in the near future.

### 3.3. Case Three

#### 3.3.1. History

A 70-year-old female with Peutz-Jeghers Syndrome (PJS) and a history of familial adenomatous polyposis had developed anemia and hematochezia. Eight years prior to this presentation the patient had undergone laparotomy for small bowel polypectomies when her surveillance endoscopies and imaging had demonstrated the presence of the polyps.

#### 3.3.2. Intervention

**Figure 5:**
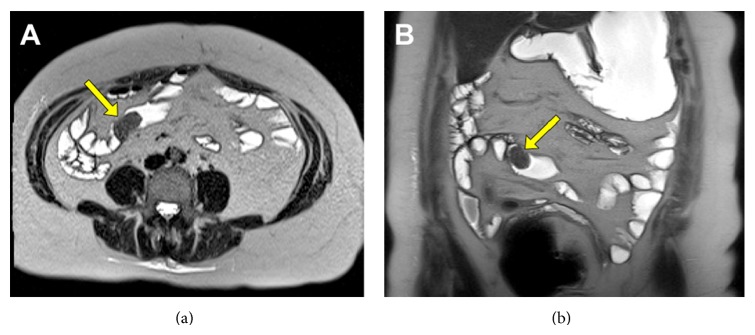
Axial (a) and coronal (b) MRE images with a large intraluminal (Peutz-Jeghers) polyp visible (yellow arrows).

**Figure 6:**
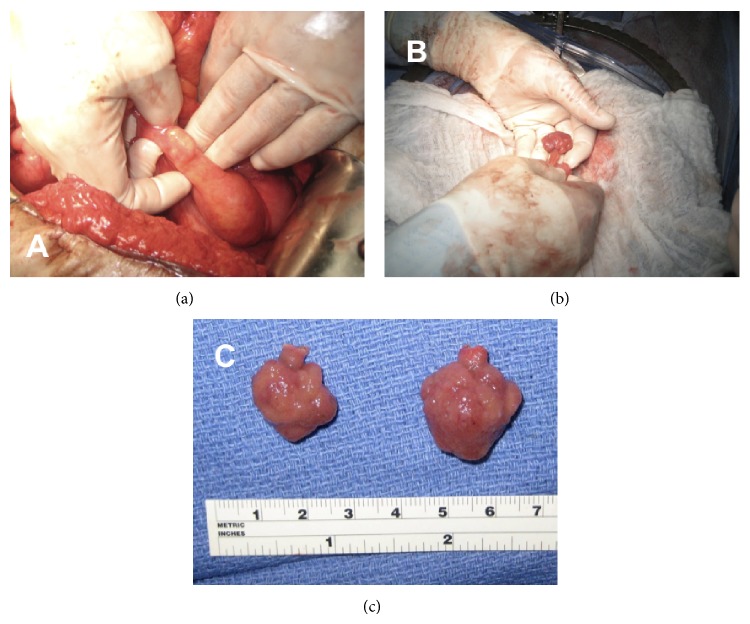
Intraoperative palpation of small bowel delineating the position of the hamartomatous polyps (a). A polyp, immediately prior to excision (b). Two Peutz-Jeghers polyps after excision (c).

The patient's semiannual enteroscopies and flexible sigmoidoscopies had not demonstrated any new polyps or other etiology for this new anemia. A capsule study was read as negative three months prior to when the MRE was done. The MRE was able to demonstrate at least seven small bowel polyps; the largest was measured at 25 mm while the smallest ones were less than 10 mm (Figure 5). The patient proceeded to laparotomy where four polyps were removed through two enterotomies in the proximal and midjejunum (Figure 6).

#### 3.3.3. Diagnosis

**Figure 7:**
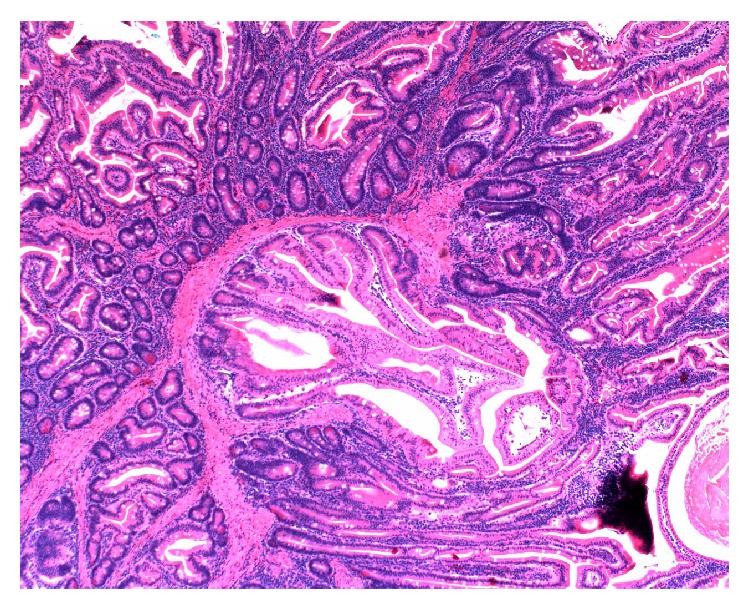
The benign adenomatous histology of one of the patient's Peutz-Jeghers polyps.

The patient had recurrent Peutz-Jeghers polyps. The pathologist confirmed that the specimens were hamartomatous polyps consistent with PJS (Figure 7). The patient is currently at home on cycled TPN due to chronic abdominal pain caused by intermittent partial small bowel obstructions. These intermittent partial small bowel obstructions are due to the fact that patient had multiple laparotomies and therefore has thick intra-abdominal adhesions.

### 3.4. Case Four

#### 3.4.1. History

A 64-year-old female with a history of left upper extremity melanoma had undergone wide local excision without sentinel lymph node biopsy one year prior to presentation to our institution. This patient then presented with 3 weeks of worsening abdominal pain with diarrhea, nausea, and vomiting.

#### 3.4.2. Intervention

**Figure 8:**
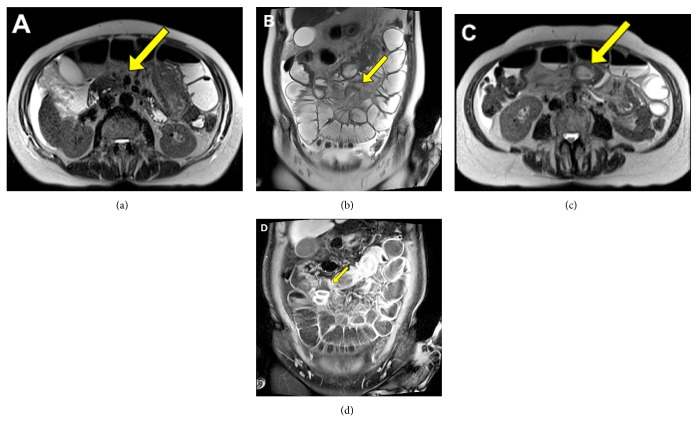
Axial (a) and coronal (b) partial-Fourier images through the pelvis show longitudinal bowel-in-bowel appearance with mesenteric fat and vessels being drawn into the intussusception. Sections through the lead point, edematous polyp, shown on axial images (c). Coronal section clearly delineates a T2 hyperintense, hypovascular, lead point (d).

The patient had a CT of abdomen and pelvis, which showed a distal ileal intussusception and incomplete high-grade small bowel obstruction. The MRE then demonstrated similar findings in addition to identifying a leading point mass that was consistent with an edematous polyp (Figure 8). The patient subsequently had an exploratory laparotomy with small bowel resection and primary anastomosis.

#### 3.4.3. Diagnosis

**Figure 9:**
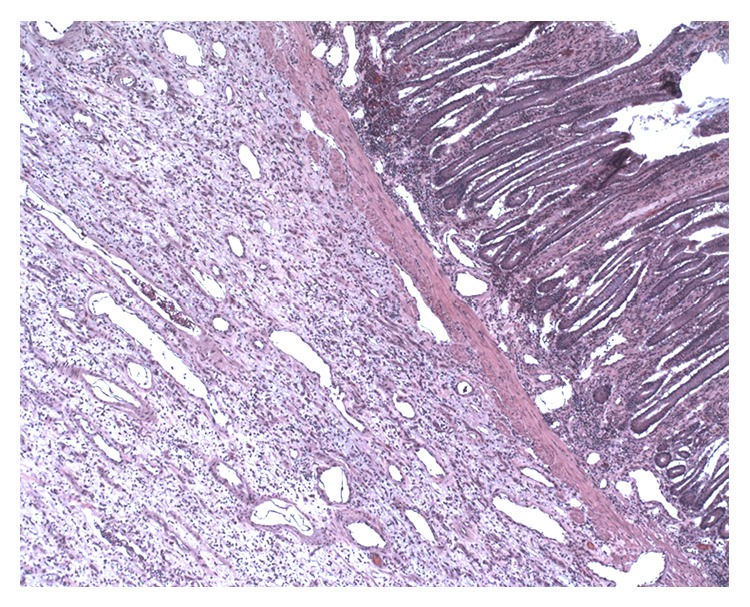
Low power of inflammatory fibroid polyp with overlying benign bowel mucosa (5x).

**Figure 10:**
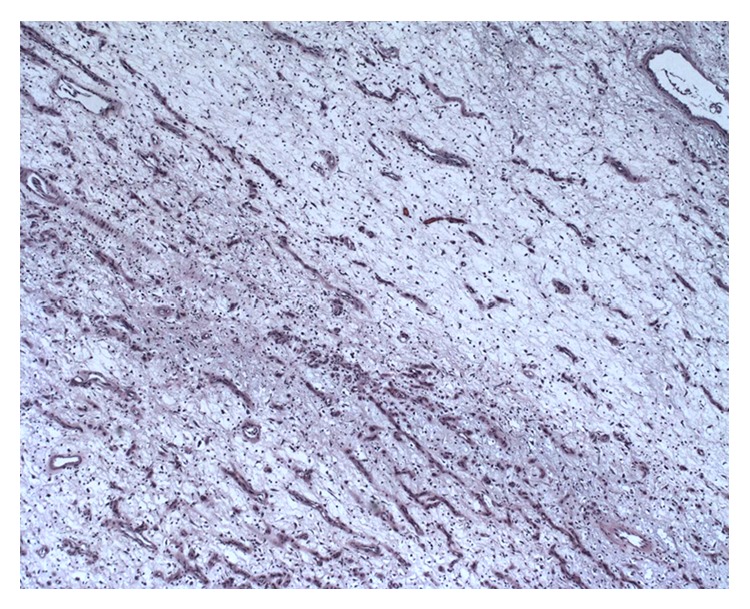
Low power of inflammatory fibroid polyp (5x). There is fibrovascular proliferation with edematous and myxoid background.

The patient had an inflammatory and edematous fibroepithelial polyp on final pathology (Figures 9 and 10). At the time of paper preparation, the patient is doing well with no complaints.

## 4. Discussion

With an experienced radiologist reading magnetic resonance enterography studies that have been obtained using a highly specialized protocol, MRE can be an exceptional investigative tool to diagnose unclear illnesses. MRE has been shown to be a useful tool in diagnosing Crohn's patients as it was found that it correlates significantly with disease activity and therefore aids in therapeutic plans [8, 9]. In addition to MRE being used in Crohn's disease, our search of literature did not produce too many additional papers that talk about its other uses. Shrot et al. looked retrospectively at MRE use at their institution over four years and have found that its most common use was in Crohn's disease, but it also was used for diagnosis of small bowel neoplasms and celiac disease [10]. It can be argued that MRE is especially useful in areas of the intestines that are difficult to access with endoscopy and in patients where visualization and biopsy of the bowel mucosa are unable to identify the underlying diagnosis, such as in the case of small bowel tumors that originate in the submucosa. We demonstrate that MRE is a useful diagnostic modality to diagnose rare colorectal disease phenomena or in patients whose surveillance is difficult, colloquially referred to as “zebras.”

In the case of patient #1, MRE was especially valuable because it made the diagnosis and the patient subsequently underwent resection of carcinoid tumor. CT and endoscopies all failed to diagnose the patient. MRE was a much more valuable diagnostic tool in this instance as it is a much more specific test. MRE can distinguish between lymph nodes and a carcinoid metastasis as the lymph nodes have higher water content and are therefore brighter. Also, soft tissue resolution of MR is better than CT. It better shows tissue planes, layers of bowel wall, and dark mesenteric strands. In addition, bowel is better distended in MRE, and wall lesions are better visualized.

In the case of patient #2, MRE was advantageous because it finally made the diagnosis, which resulted in appropriate treatment of the patient's rectal lymphoma. As already mentioned, MR shows layers of bowel wall better than CT, therefore showing better soft tissue resolution. In this case, lymphoma penetrated bowel wall and lost stratification compared with inflammatory process. Although CT would be diagnostic for lymphoma, in this instance pelvic disease is better diagnosed with MR due to volume averaging on CT.

In the case of patient #3, MRE was an exceptionally superior test because it was able to diagnose small bowel polyps in a patient with Peutz-Jeghers Syndrome when enteroscopy, flexible sigmoidoscopy, and small bowel capsule study were all negative for any new polyps. Polyps are better distinguished on MR because biphasic lumen is bright on T2 and dark on T1 images. They stand out against water on T2 and enhance on T1 as a bright filling defect within the lumen. Also, MR is very sensitive to contrast enhancement compared with CT. Few molecules of gadolinium are very bright on MRE. It takes only a few milliliters of contrast to show up as increased density. The problem with CT is that mucosa enhances significantly which results in tumor enhancement not being conspicuous. Therefore, MR is superior for soft tissue resolution. In MRE, bowel lumen is better distended while, in CT images given with oral contrast, which is a cathartic and often promotes peristalsis, collapsed bowel can mask pathology. It is possible to distend bowel with CT, but if not distended enough, then more oral contrast would have to be given and CT repeated, which would result in double radiation. With MRE, psyllium promotes expansion with intralumenal liquid. Furthermore, even if the bowel is not distended enough, more psyllium can be given and the patient can be reimaged without additional ionizing radiation.

In the case of patient #4, MRE was helpful as it clearly showed that the reason for intussusception and small bowel obstruction was an edematous polyp rather than a melanoma metastasis, which is one of the most common etiologies of small bowel tumors in a patient with a history of melanoma [11]. While melanoma is one of the most common metastatic lesions to the small bowel in patients with a prior history of melanoma, the most common small bowel primary neoplasms are adenocarcinoma, carcinoid, lymphoma, and GIST. There are some typical imaging findings on CT and MRI that distinguish these lesions from one another. Adenocarcinoma presents as concentric lumen narrowing with irregular edges. Carcinoid appears as a small filling defect. Lymphoma can be seen as coarse segmental wall thickening with ulceration and necrosis, while GIST appears as larger regular mass with nonhomogenous enhancement. Metastases, on the other hand, present as solid intraluminal nodules developing from hematogenous spread to submucosal layers [12]. However, our patient was found to have a lesion with homogeneous enhancement which caused intussusception and obstruction. Inflammatory fibroepithelial polyps are benign masses that can occur anywhere along the gastrointestinal tract. As they occur very rarely in the small bowel, there are case reports describing them causing intussusception and small bowel obstruction [13, 14]. Again, MRI has better distention and soft tissue resolution, which result in better visualization of endolumenal masses. In addition, MRI has fat-saturated sequence. An edematous polyp as a lead point will be bright on T1 and T2 TSE, while a solid tumor with higher malignant potential would not. When fat-saturated sequences are obtained, if lead point loses signal, the study becomes diagnostic of a fat-containing lesion, like a lipoma. The fat-saturated sequences were negative on this patient, suggesting it was not a lipoma, but rather, another, benign tumor. This resulted in patient reassurance and subsequent surgical resection.

There are limitations to the use of MRE, and it is important to note that MRE costs more than ultrasound or CT scan. Although it gives a three-dimensional view, it does not differentiate inflammatory from infectious colitis, benign from malignant strictures, and neoplasia from carcinoma and therefore should be used as an adjunct tool to define unclear pathology. Magnetic resonance is limited in patients who suffer from claustrophobia, although “open MR” machines have improved on this. MRE should be judiciously used in patients with decreased renal function (glomerular filtration rate GFR <50 cc/min) due to the risk of nephrogenic systemic fibrosis with gadolinium administration.

In conclusion, we believe that MRE, under the protocol described in this paper, is the test of choice in patients whose gastrointestinal surveillance is difficult or when used to diagnose rare GI disease phenomena, colloquially referred to as “zebras.”

## Figures and Tables

**Table 1:** Summary of the four patients.

Patients	Age/gender	Studies	MRE	Diagnosis	Treatment
#1	53/male	Endoscopies, small bowel enteroscopy, CT	Mass in the small bowel mesentery consistent with carcinoid	Carcinoid in the distal ileum with invasion into the mesentery	Exploratory laparotomy with resection

#2	34/male	Colonoscopies	Rectal lymphoma with sever but nonobstructing luminal narrowing	Rectal lymphoma in HIV patient	Chemo

#3	70/female	Enteroscopies, flexible sigmoidoscopy, capsule study	7 small bowel polyps	Recurrent Peutz-Jeghers	Exploratory laparotomy with excision of polyps

#4	64/female	CT abdomen/pelvis	Suspected edematous polyp as a leading point of intussusception and small bowel obstruction	Inflammatory fibroid polyp causing intussusception	Exploratory laparotomy with resection and primary anastomosis
